# Supplementary material for: Challenges and Opportunities of Male Partner Involvement in Cervical Cancer Prevention and Control in Central Kenya: A Qualitative Analysis
Source: Int J Environ Res Public Health. 2025 Oct 15;22(10):1575. doi: 10.3390/ijerph22101575 (PMC12564609; doi:10.3390/ijerph22101575)
Supplement: Supplementary file 1 [file ijerph-22-01575-s001.zip › ijerph-3752343-supplementary.pdf]

Table S1. Male Partner Involvement in Cervical Cancer Prevention and Control Codebook.

Social Ecological Model (SEM)	Code	Definition	Illustrative Quote
Individual	Limited Knowledge about Cervical Cancer	Apply this code to Gaps in awareness or misconceptions about cervical cancer, HPV, or prevention methods.	*"Honestly, not much. I have heard it's a woman sickness, but I don't know how it happens."*
	Financial Support	Apply this code to Men providing money to support partners' access to screening or treatment.	*"Sometimes it is difficult to go to hospital when you don't have money but if you need to do a test for cancer, he gives you money and you feel comfortable to go."*
Interpersonal	Emotional and Accompaniment Support	Apply this code to male partner emotional and physical support such as encouragement or accompanying women to the clinic or support in domestic work.	*"I would appreciate if my husband accompanies me to the clinics, but he always says there is no need as I am not sick."*
	Communication about Sexual Health	Apply this code to open dialogue between partners on HPV, sexual health, and prevention.	*"Men and women should discuss about their sexual health even if it is a sensitive topic."*
Community	Community Perceptions and Stigma	Apply this code to social norms discouraging male involvement in reproductive health.	*"Some communities see reproductive health as a woman's secret. Men fear being mocked if they care 'too much.'"*
	Role of Community Leaders	Apply this code to Influence of religious, cultural, or local leaders in promoting male involvement.	*"Men listen to other men. If local leaders, like pastors, coaches, or elders, talk about it, more will take it seriously."*
Health System	Health Worker Attitudes	Apply this code to Positive or negative experiences with health providers that influence male participation.	*"Clinics don't always welcome men. They focus only on women."*

	Clinic Environment and Organization	Apply this code to physical space, waiting times, and service delivery affecting men’s willingness to attend health facilities.	*“I felt so uncomfortable. They should expand the space or the sitting area to avoid inconveniences.”*
	Couple-focused Education	Apply this code to educating both men and women together on prevention, screening, and vaccination.	*“If health workers educated couples together, more men would participate.”*
Policy/Structural	HPV Vaccination Policy	Apply this code to perceptions of vaccination programs for boys and girls.	*“And include HPV vaccination for boys in national programs. If it’s only for girls, men won’t see themselves as part of it.”*
	Workplace and Policy Support	Apply this code to Institutional policies that allow men to accompany partners.	*“Yes! Like workplace leave for men to take their partners to screenings.”*
	Accessibility and Affordability	Apply this code to ease of access of services or ability to afford or not afford services.	*“Mobile clinics in workplaces or markets could help. Bring services where men already are.”*
